# *Anaplasma phagocytophilum *- pathogen with a zoonotic potential

**DOI:** 10.1186/1756-3305-7-S1-O24

**Published:** 2014-04-01

**Authors:** S Stuen, EG Granquist, C Silaghi

**Affiliations:** 1 Department of Production Animal Clinical Sciences, Norwegian University of Life Sciences Sandnes, Norway; 2 Department of Production Animal Clinical Sciences, Norwegian University of Life Sciences, Oslo, Norway; 3 Department of Veterinärwissenschaftliches,Comparative Tropical Medicine and Parasitology, Ludwig-Maximillians-Universität München, Munich, Germany

## 

*Anaplasma phagocytophilum *has for decades been known to cause the disease tick-borne fever (TBF) in domestic ruminants in *Ixodes ricinus*-infested areas in northern Europe. Later studies have shown that the infection is widespread on the continent. *A. phagocytophilum *is able to persist between seasons of tick activity in several mammalian species and movement of hosts and infected ticks on migrating animals or birds may spread the bacterium. *A. phagocytophilum *encompasses multiple genetic strains, a broad host range and has pathogenic potential in several other mammalian species, including humans. Identification and stratification into phylogenetic subfamilies has been based on cell culturing, experimental infections, PCR and sequencing techniques. The clinical symptoms vary from subclinical to fatal conditions, and considerable underreporting of clinical incidents is suspected in both human and veterinary medicine. In human, the most common clinical manifestations are flu-like symptoms two to three weeks after tick attachment. In Europe, human granulocytic anaplasmosis (HGA) was first described in 1997. Since then only few cases of HGA have been reported. The reason for the suspected underreporting may be lack of awareness among physicians, unspecific clinical signs, lack of available adequate hosts and non-virulent strains of *A. phagocytophilum *involved. Phylogenetic studies indicate that stains isolated from humans, dogs and horses from Europe belongs to the same clonal complex, while similar studies on isolates from wild and domestic ruminants indicate that they are unlikely to harbour variants of *A. phagocytophilum *that are infectious to humans. In contrast, wild boar and hedgehogs are suspected to be competent reservoir hosts for human variants. However, the natural transmission cycles of various *A. phagocytophilum *strains, the involvement of their respective hosts and vectors, and in particular their zoonotic potential, have to be unravelled. Updated information concerning the zoonotic potential of *A. phagocytophilum *will be presented.

